# Supplementary material for: Seminoma and Embryonal Carcinoma Footprints Identified by Analysis of Integrated Genome-Wide Epigenetic and Expression Profiles of Germ Cell Cancer Cell Lines
Source: PLoS One. 2014 Jun 2;9(6):e98330. doi: 10.1371/journal.pone.0098330 (PMC4041891; doi:10.1371/journal.pone.0098330)
Supplement: File S1 — ZIP file containing DMRforPairs output for significant regions. Please start from the html files. (ZIP) [file pone.0098330.s008.zip › figures/20998.pdf]

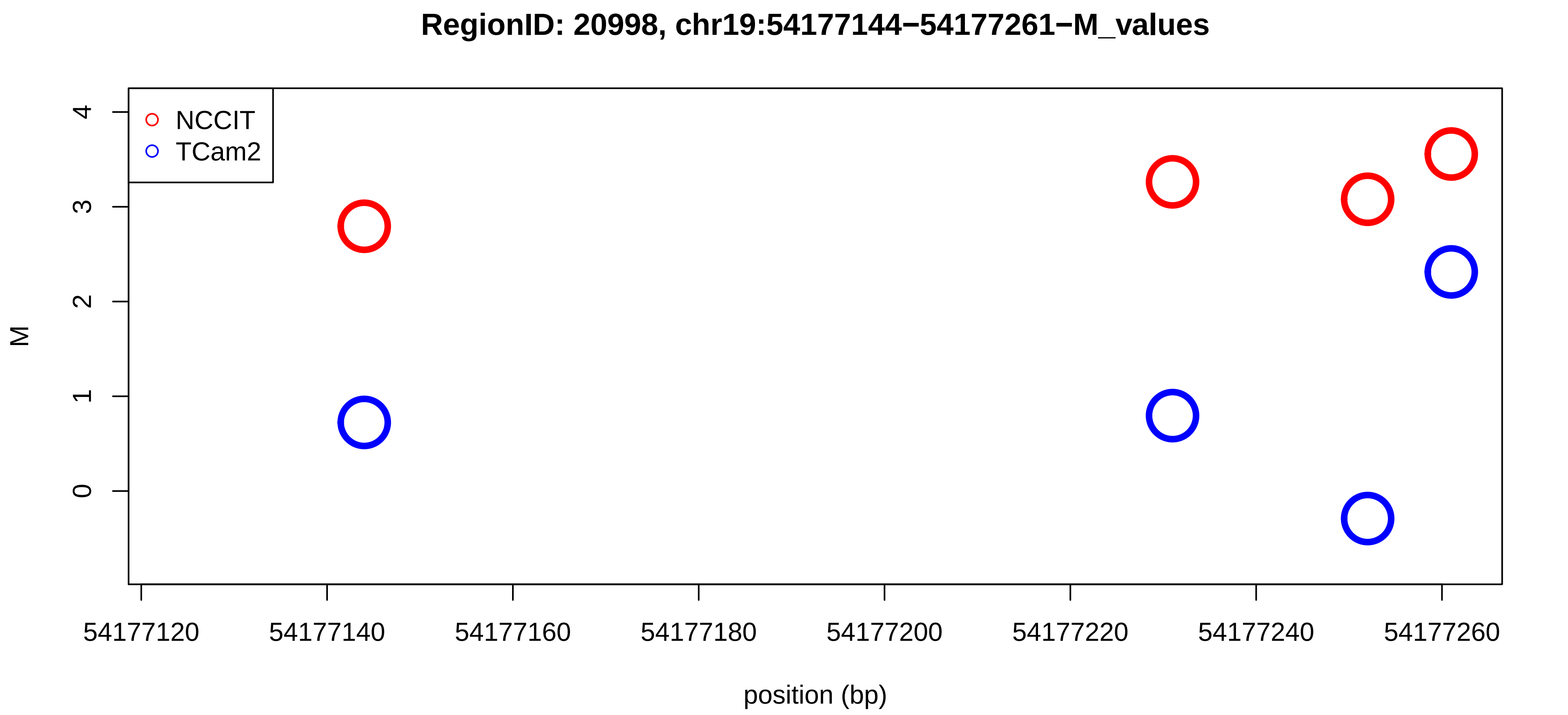

RegionID: 20998, chr19:54177144−54177261−M_values
NCCIT
TCam2
M
0
1
2
3
4
54177120
54177140
54177160
54177180
54177200
54177220
54177240
54177260
position (bp)

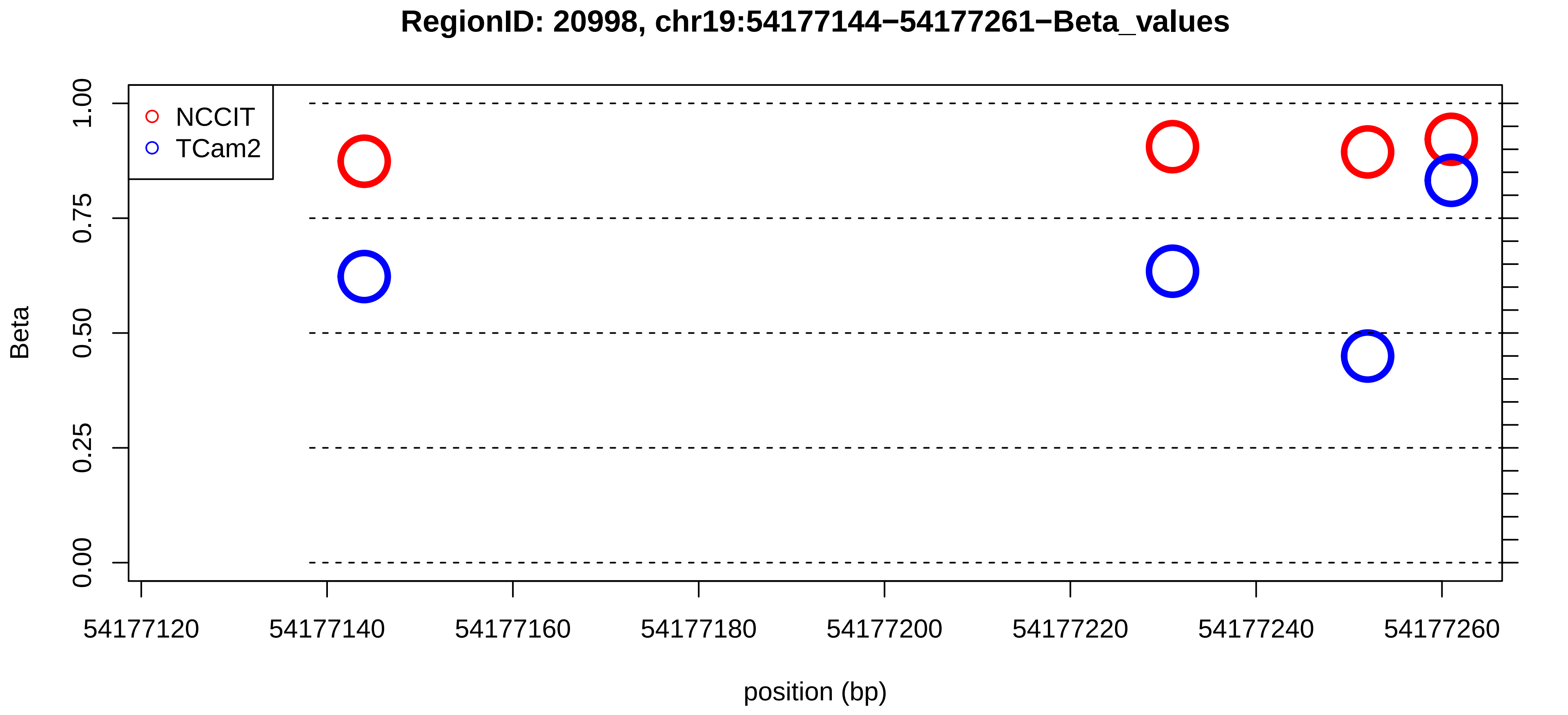

RegionID: 20998, chr19:54177144−54177261−Beta_values
NCCIT
TCam2
Beta
0.00
0.25
0.50
0.75
1.00
54177120
54177140
54177160
54177180
54177200
54177220
54177240
54177260
position (bp)
